# Permutation – based statistical tests for multiple hypotheses

**DOI:** 10.1186/1751-0473-3-15

**Published:** 2008-10-21

**Authors:** Anyela Camargo, Francisco Azuaje, Haiying Wang, Huiru Zheng

**Affiliations:** 1 University of East Anglia, School of Computing, Norwich, NR4 7TJ, England, UK; 2 Laboratory of Cardiovascular Research, CRP-Santé, L-1150, Luxembourg; 3 University of Ulster at Jordanstown, School of Computing and Mathematics, Shore Road, Newtownabbey, Co. Antrim, BT37 0QB, Northern Ireland, UK

## Abstract

**Background:**

Genomics and proteomics analyses regularly involve the simultaneous test of hundreds of hypotheses, either on numerical or categorical data. To correct for the occurrence of false positives, validation tests based on multiple testing correction, such as Bonferroni and Benjamini and Hochberg, and re-sampling, such as permutation tests, are frequently used. Despite the known power of permutation-based tests, most available tools offer such tests for either *t*-test or ANOVA only. Less attention has been given to tests for categorical data, such as the Chi-square. This project takes a first step by developing an open-source software tool, Ptest, that addresses the need to offer public software tools incorporating these and other statistical tests with options for correcting for multiple hypotheses.

**Results:**

This study developed a public-domain, user-friendly software whose purpose was twofold: first, to estimate test statistics for categorical and numerical data; and second, to validate the significance of the test statistics via Bonferroni, Benjamini and Hochberg, and a permutation test of numerical and categorical data. The tool allows the calculation of Chi-square test for categorical data, and ANOVA test, Bartlett's test and t-test for paired and unpaired data. Once a test statistic is calculated, Bonferroni, Benjamini and Hochberg, and a permutation tests are implemented, independently, to control for Type I errors. An evaluation of the software using different public data sets is reported, which illustrates the power of permutation tests for multiple hypotheses assessment and for controlling the rate of Type I errors.

**Conclusion:**

The analytical options offered by the software can be applied to support a significant spectrum of hypothesis testing tasks in functional genomics, using both numerical and categorical data.

## Background

Current statistical inference problems in areas such as genomics and proteomics regularly involve the simultaneous test of hundreds of null hypotheses. This strategy has allowed scientists to unveil important cues on the mechanisms involved in the development of deadly diseases. For example, Barth et al. (2006) [1] analysed gene expression patterns related to dilated cardiomyopathy (DCM) and identified specific gene regulatory relationships relevant to this disease condition. By means of Significant Analysis of Microarray (SAM) and Nearest Shrunken Centroid (NSC), 27 genes, whose expression profiles were sufficient to differentiate between DCMs and non-failing hearts samples, were identified. Mathur et al. (2005) [2] analysed antibody arrays and identified potential candidates for ischemic preconditioning-associated vascular growth pathways. Potential candidates were identified by applying a cut-off threshold value that filtered out non-significant probes. When dealing with these and related types of data, many hypotheses are tested and each test has a specified Type I (i.e. false positive) error probability, which is associated with the chance of committing Type I errors [3]. Therefore, it is important to define an appropriate Type I error threshold, as well as selecting an effective multiple testing procedure to control this error rate and account for the joint distribution of the test statistics.

To correct for the occurrence of false positives, validation tests based on multiple testing corrections and re-sampling techniques (i.e. permutation-based test) are frequently used. Although both strategies aim to control Type I error, these techniques implement different approaches to estimating errors and rejecting null hypotheses. Traditional multiple-testing corrections, such as Bonferroni and variations, adjust P-values derived from multiple statistical tests to correct for the occurrence of false positives [4]. The Benjamini and Hochberg (B&H) ranks P-values in an ascending order, multiplies them by the number of features, and divides them by their corresponding rank [5]. The permutation test re-samples N times the total number of observations, in a population sample, to build an empirical estimate of the null distribution from which the test statistic has been drawn [6]. In the end, the application of these methods leads to either the rejection or acceptance of the null hypothesis. The Bonferroni correction is known to be extremely conservative. It can lead to Type II (i.e. false negative) errors of unacceptable levels, which may contribute to publication bias and the exclusion of potentially relevant hypotheses (e.g. significant differential expression between patient groups or genotype-phenotype associations) [7]. In contrast, B&H is less stringent, which may lead to the selection of more false positives [5]. Unlike Bonferroni and B&H, permutation tests do not use individual association scores based on family-wise corrections [8]. Instead, permutation-based tests estimate statistical significance directly from the data being analysed. More importantly, irregularities of the observed data are maintained in the permuted data sets and are included in the estimation of the permutation probability [9].

To date, permutation tests have become widely accepted and recommended in studies that involved multiple statistical testing [3,6,7]. Despite its power, current available tools, such TIGR MeV [10], offer permutation tests to estimate P-values for either *t*-test or ANOVA only. Another example is GeneSpring [11] that offers a permutation test for multiple testing for either *t*-test or ANOVA test statistics only. These and other tools published do not offer multiple-testing solutions for categorical data, such as the Chi-square. This test is appropriate for the analysis of SNPs (single nucleotide polymorphisms) data to identify significant patterns of genetic variability, i.e. variation-phenotype associations. Another important statistical significance assessment technique not available in well-known open-source tools is the Bartlett test, which may be used for testing equality of variances or the significance of data dispersion differences across groups. Moreover, the Bartlett test should also be used before attempting the calculation of either ANOVA or *t*-test, as they assume that variances are equal across groups or samples.

**Figure 1:**
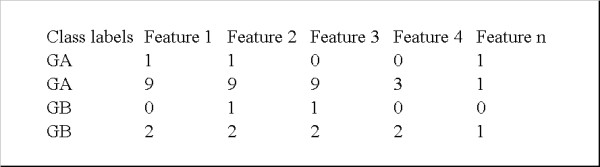
**Format specifications of the input data**. Format specifications of the input data. Rows represent samples and columns represent features.

Given the evident need to offer software tools incorporating such statistical tests with options for correcting for multiple tests, this study takes a first step by developing a public-domain, user-friendly software with the following functionality. The tool allows the calculation of Chi-square test for categorical data, ANOVA test, Bartlett's test and *t*-test for paired and unpaired data. Once a test statistic is calculated, Bonferroni, B&H and a permutation tests are implemented, independently, to control for Type I errors. P-values from the permutation test were estimated as follow, using the data encoding format shown in Figure 1:

First, test statistic and corresponding P-value are calculated on the original data set. Data are permuted at random *B *times and test statistics are calculated on each permuted data set. Third, permuted distribution is calculated by: counting the times (K) the statistic value obtained in the original data set was smaller than the statistic value obtained from the permuted data sets, and dividing that value by the number of random permutations i.e. K/*B*. Results are stored in a text file for subsequent analyses. Table 1 offers guidelines for the selection of the most appropriate statistical test under this system.

**Table 1:** Statistical tests provided by the Ptest software.

**Goal**	**Measure**	**Data type**	**Test**
To compare two unpaired groups	Mean	Numerical	unpaired *t*-test
To compare two paired groups	Mean	Numerical	paired *t*-test
To compare two or more unmatched groups	Proportions	Categorical	Chi-square test
To compare two groups	Variance	Numerical	Bartlett
To compare three or more unmatched group	Mean	Numerical	ANOVA

Selecting the correct statistical test available in the Ptest software.

## Implementation

The software is a Java-based, command-line tool [see Additional files 1 and 2]. Input data are presented in a plain text file, where rows represent samples and columns represent features (Figure 1). The maximum number of groups to be compared is two, with two exceptions: the Chi-square test, for categorical data, and the ANOVA test for numerical data, which permit the comparison of more than two more groups. These requirements have been defined because they cover most of the typical multiple-testing applications in gene expression and SNPs data analysis. New functionality (e.g. Windows interface or other relevant tests) could be added based on future requirements and additional external user feedback.

### Statistical tests

The tool has at the user's disposal the following statistic tests: Student's test for numerical data, two classes; Bartlett's test for numerical data, two classes; ANOVA test for numerical data, more than three classes; and Chi-square for categorical data, two or more classes. For detailed information about each test, please refer to National Institute of Standards and Technology/Semiconductor Manufacturing Technology e-Handbook of Statistical Methods [12].

### Multiple hypotheses testing procedures

Given (*N*) number of samples, (*C*) number of classes, (*F*) number of features, (*S*) significance level, (*B*) number of permutations, and (*T*(*obs*)) test statistic, validation methods are as described bellow:

Multiple testing correction: P-values, according to test statistic and degrees of freedom (N-2), were obtained and adjusted under Bonferroni and B&H multiple testing corrections B[5]. Permutation test: test statistic is estimated from original data set *T*(*obs*); sample's labels are shuffled *B *times and *T*(*obs*)s' are obtained; if *T*(*obs*) <*T*(*obs*)' a counter *T*(*per*) is increased by 1. The probability that *T*(*obs*) occurred by chance alone is: *T*(*per*)/*B*.

### Software usage

**Figure 2:**
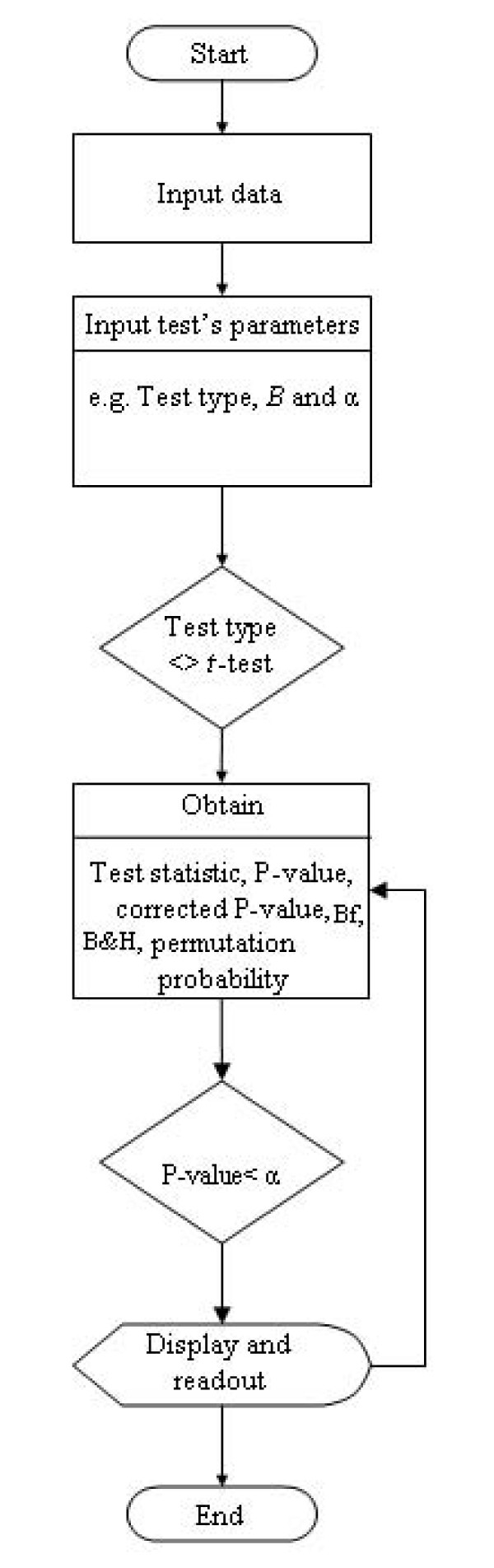
**Permutation test tool usage flowchart**. Permutation test tool usage flowchart. Significance level (α), number of permutations (*B*). Bonferroni (Bf) and Benjamini and Hochberg (B&H).

Typical usage involves a user providing the following information: file name containing the data to be analysed, the name of new file where results are to be stored, the selection of test statistic to be calculated, the significance level at which the null hypothesis is to be rejected, and the number permutated data sets to be created for the estimation of the null-hypothesis distribution (Figure 2) [see Additional file 3]. Depending on the test statistic to be calculated, the user may need to provide additional information in a few steps. For example, if the *t*-test is selected, the user should indicate whether samples (i.e. groups being compared) are independent or not (paired). The user is also allowed to specify which type of distribution should be used: one- or two-tailed distribution. Once the required information is provided, the tool performs the analysis and displays those features whose raw P-values are below the significance level, their corrected P-value after Bonferroni correction, their corrected P-value after B&H correction, and their corrected P-values after performing the permutation test.

**Figure 3:**
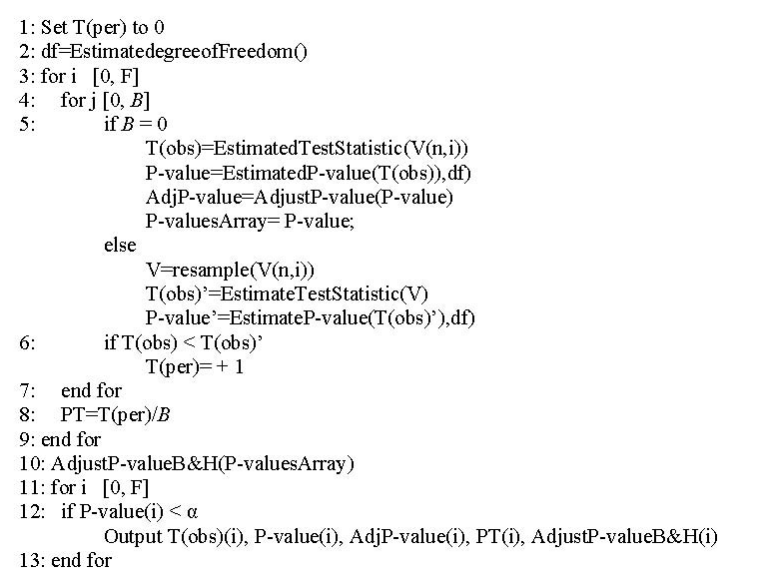
**Algorithm for multiple testing correction based on permutation test**. Algorithm for multiple testing correction based on permutation test. Significance level (α), number of permutations(*B*), counter (T(per)), number of features (F), test statistic original data (T(obs)), test statistic permutated dataset (T(obs)').

Figure 3 is a pseudo-code representation of the multiple testing correction procedure implemented.

## Results

To illustrate some of the advantages of using the permutation-based test for multiple hypotheses validation, this section summarises examples of analyses using publicly available data. This includes a comparison with results obtained when Bonferroni correction was applied (Table 2).

**Table 2:** Results of analyses of statistical tests.

**Test**	**Data description**	**Groups**	**Features**	**Samples**	**Feature selection according to**			
					
					**raw P-value**	**Multiple test correction**		
						
						**Bonferroni**	**B&H**	**PT**
Bartlett	Microarray Numerical	2	8068	12	526	1	1	327
*t*-test	Microarray Numerical	2	8068	12	1413	2	39	1398
Chi-square	Single nucleotide polymorphisms (SNP) Categorical	3	334	33	153	8	131	153
ANOVA	Microarray Numerical	3	14976	37	6371	9	3331	6262

Comparative statistics when multiple-testing correction is based on either Bonferroni correction, Benjamini and Hochberg (B&H), or permutation test (PT), against potentially significant features (P-value) before correction. Significance level (α) = 0.05, Number of permutations (*B*) = 10000.

### Testing data sets

Three data sets were used in the analysis:

1) A microarray data set generated by a study in dilated cardiomyopathy was obtained from the GEO (Gene Expression Omnibus) [13], accession number GDS2205 (for numerical data analysis) and composed of 12 samples: 5 from non-failing hearts and 7 from DCM patients.

2) A genotype data set (for categorical data analysis) was obtained from the Single Nucleotide Polymorphism database (SNPdb) [14]. This data set was composed of 34 samples, 10 from African-American people, 12 from European-American people and 11 from Han-Chinese people.

3) A microarray data set, oligo array, generated by a study in heart failure was obtained from the GEO, accession number GDS1362, was composed of 37 samples: 7, 20 and 10 samples were obtained from non-failing hearts, DCM heart, and Ischemic cardiomyopathy (ICM) patients respectively.

### Data pre-processing

Microarray data: probe sets with absent calls in more than 50% of their transcripts were discarded. Transcripts of probe sets corresponding to similar gene symbols were averaged. Data were normalised per chip and then per gene. Values were transformed using the mean and standard deviation of the row (per gene) or column (per chip). Genotype data did not require pre-processing.

### Statistical analyses

The first analysis calculated Bartlett's test statistic to determine whether the variances of two experimental groups, from a microarray data set [see Additional file 4], were equal. The null hypothesis of this analysis was that there was no significant difference between the variances of the two groups, and the significance level to reject the null hypothesis was set to 0.05. Data set was composed of 12 samples: 5 and 7 samples were obtained from non-failing hearts and DCM patients respectively. Out of 8068 genes, 526 genes were found to be statistically significant (P < 0.05, before correction for multiple-testing), one gene was under the significance level after correcting with Bonferroni, and one gene was under the significance level after correcting with B&H. However, after performing the permutation test, 327 genes were found significantly differentially expressed (P < 0.05). That is, the two group samples being compared exhibit equal variances, which is commonly expected in typical microarray data analyses.

The second analysis implemented the *t*-test (type: two sample equal variances; number of distribution tails: two-tailed): equal variances and two tailed) to estimate the potential statistical significant difference between the means of two (normally distributed) experimental groups, from the same microarray data set analysed above [see Additional file 5]. The null hypothesis of this analysis was that there was no difference between the means of the two groups, and the significance level to reject the null hypothesis was set to 0.05. In this case, the raw P-values of 1413 genes were under the significance level (P < 0.05), 39 genes were under the significance level after correcting with B&H, and only two genes were under the significance level after correcting with Bonferroni. In this case, results were consistent with our expectations: B&H identified more genes than Bonferroni did, which shows that the former tends to be less stringent. After performing the permutation test, 1398 genes were found significantly differentially expressed (P < 0.05). In addition, we noted that the raw P-values of some of the genes filtered out by Bonferroni were well below the significance level, i.e. they were potentially significant under a less conservative correction approach. For example, raw P-values of ACVR1 and CFHR1 were 0.0004 and 0.004, respectively, and their P-values after Bonferroni correction were above 0.9. However, based on the permutation-based test, these two genes fall below the significance threshold (corrected P values: 0.0001 and 0.001 for ACVR1 and CFHR1, respectively). This, as expected, shows the statistical power of permutation-based procedures for multiple testing.

The third analysis implemented the Chi-square test on categorical data derived from a genetic variation data set (SNPs) [see Additional file 6]. The problem was to determine statistically significant genetic variations among the SNPs of three ethnic groups: African-American, European-American and Chinese. The data encode genotype values for each SNP under each group [15]. This data set was composed of 34 samples: 10 from African-Americans, 12 from European-Americans and 11 from Han-Chinese people. The null hypothesis of this analysis was that there were no genetic differential variations among the three groups, and the significance level to reject the null hypothesis was set to 0.05. In this case the raw P-values of 153 SNPs, out of 334, were under the significance level (P < 0.05). Bonferroni correction identified only eight SNPs, whose P-values were below significance level, and B&H correction identified 131 SNPs, whose P-values were below significance level. In contrast, the permutation test identified more features than B&H: 153 SNPs with significant P-values. These results are consistent with the results reported by Carlson, et al. (2003) [16], which found that only 48% of the SNPs were shared by African-Americans and European-Americans. In our study, the permutation-based adjustment found that 55% of SNPs showed no significant differences among the three populations been analysed. These results again confirm the statistical power of permutation-based procedures for multiple testing.

A fourth analysis implemented the ANOVA test to estimate the potential statistical significant difference between the means of three (normally distributed) experimental groups. Samples in this data set were obtained from heart tissue of healthy donors, as well as from donors suffering from either dilated or ischemic cardiomyopathy [see Additional file 7]. We used the ANOVA test to look for possible outstanding differences among the three populations evaluated, because *t*-test is designed to perform pair-wise comparisons, only. The null hypothesis of this ANOVA analysis was that there were no differences between the means of the three groups, and the significance level to reject the null hypothesis was set to 0.05. In this case, the raw P-values of 6371 genes were under the significance level (P < 0.05), 3331 genes were under the significance level after correcting with B&H, and only nine genes were under the significance level after correcting with Bonferroni. After performing the permutation test, 6262 genes were found significantly differentially expressed (P < 0.05). The genes reported as significantly differentially after correcting via Bonferroni were not included in the set of potentially significant genes detected by the permutation test. In addition, we compared our results against those previously reported by Kittleson, et al. (2005) [17] and found that most genes reported by them as significantly differentially expressed were also below significant level when our permutation test was performed, or when P-values were corrected via the B&H method. In contrast, only one of the genes reported by Kittleson's was also below significant level after we corrected with Bonferroni. Perhaps this analysis showed the real strength that the permutation test has to identify potential biomarkers of disease.

## Conclusion

The techniques for multiple testing offered here through a platform-independent tool are relevant to a variety of data analysis tasks in biology and medicine. The results also allowed us to illustrate the power of a permutation test for multiple hypotheses assessment procedures and for controlling the rate of Type I errors. We also demonstrated that even when P-values were corrected via B&H, which is considered a less stringent method as opposed to Bonferroni, a number of potentially significant features were dismissed. The software is easy to use and it offers the basis for future extensions. Another key contribution is the implementation of multiple hypotheses statistical testing techniques for both numerical and categorical data. The analytical options offered can be applied to support a significant spectrum of hypothesis testing tasks in functional genomics, e.g. fast detection of significantly differentially expressed genes and genotypes. Moreover, to the best of our knowledge, this is the first open-source software tool freely available for supporting less traditional genomic applications, such as the detection of between-group differences on the basis of SNPs. In this area multiple-testing procedures have traditionally relied on very stringent adjustment approaches (e.g. Bonferroni).

Despite its simplicity, in terms of usability, this tool in comparison with others, such as GeneSpring and TIGR MeV, offers the following advantages: Freely-available, as TIGR MeV does, no computational installation cost, easy to use, computationally inexpensive. Moreover it allows the calculation of traditional statistical tests and multiple testing with categorical data, as well as test- and distribution-independent permutation-based tests.

We expect to continue expanding the tool with alternative statistical significance measures, such as Fisher's exact test, Z or Wald scores. We will welcome additional user's feedback after the publication of this article.

## Availability and requirements

**Project name: **Permutation-based statistical tests for multiple hypotheses

**Project home page: **

**Operating system(s): **Platform independent

**Programming language: **Java

**Other requirements: **Java 1.5.1 or higher

**License: **None

**Any restrictions to use by non-academics: **None

## Abbreviations

ANOVA: Analysis of variance; DCM: Dilated CardioMyopathy; SAM: Significant Analysis of Microarray; NSC: Nearest Shrunken Centroid; SNP: Single nucleotide polymorphisms

## Competing interests

The authors declare that they have no competing interests.

## Authors' contributions

AC co-designed the software, wrote and implemented all source code, co-evaluated their outcomes and co-wrote the manuscript. FA conceived the original study, contributed to the testing and evaluation phases, and co-wrote the manuscript. HW co-designed the software, co-evaluated their outcomes and co-wrote the manuscript. HZ co-designed the software, co-evaluated their outcomes and co-wrote the manuscript. All authors read and approved the final manuscript.

## Supplementary Material

Additional file 1 **Ptest software**. Execute from command line like this\java Ptest. Click here for file

Additional file 2 **EasyInput library**. Used by Ptest. Click here for file

Additional file 3 **README**. Microsoft word file, it should be open with Microsoft word because it contains mathematical equations. Click here for file

Additional file 4 **Example data set for Bartlett's test**. 2 classes, 12 samples, 5 and 17 respectively, 8068 features. Plain text file can be open with any word processor. Click here for file

Additional file 5 **Example data set for  t-Test – unpaired samples**. 2 classes, 12 samples – 5 and 17 respectively – 8068 features. Plain text file can be open with any word processor. Click here for file

Additional file 6 **Example data set for Chi-square test – categorical data**. 3 classes, 33 samples – 10, 12, 11 samples respectively – 334 features. Plain text file can be open with any word processor. Click here for file

Additional file 7 **Example data set for ANOVA test.** 3 classes, 37 samples – 7, 20 and 10 respectively – 14976 features. Plain text file can be open with any word processor. Click here for file
