# Supplementary material for: Permutation – based statistical tests for multiple hypotheses
Source: Source Code Biol Med. 2008 Oct 21;3:15. doi: 10.1186/1751-0473-3-15 (PMC2611984; doi:10.1186/1751-0473-3-15)
Supplement: Additional file 3 — README. Microsoft word file, it should be open with Microsoft word because it contains mathematical equations. [file 1751-0473-3-15-S3.rtf]

Permutation-based statistical tests for multiple hypotheses 

Introduction
=========
We developed a public-domain, user-friendly software whose purpose was twofold: first, to estimate test statistics for categorical and numerical data; and second, to validate the significance of the test statistics via Bonferroni and a permutation test of numerical and categorical data. The tool allows the calculation of Chi-square test for categorical, ANOVA test, Bartlett's test and t-test for paired and unpaired data. Once a test statistic is calculated, Bonferroni, Benjamini and Hochberg (B&H), and a permutation tests are implemented, independently, to control for Type I errors. In the end, the tool displays statistical details of those features whose P-values were below the significance level defined by the user. The results obtained from the analysis of public data illustrated the power of a permutation test for multiple hypotheses assessment procedures and for controlling the rate of Type I errors.

Installation
========
Unzip the file in a directory of your preference

Input data
========

Provide a .txt file with the following structure: First column identifying class labels, first row identifying features label. Data should be allocated within these two set of labels. 

Class labels	Feature 1	Feature 2	Feature 3	Feature n	
GA                	1                	0                	1		0 
GA                	1                	0               	1 		1
GB                	1                	0                	1 		2
GB                	1                	0                	1 		1

** Rows represent samples and columns represent features.

Software algorithm
==============

Let V be an array of n samples and n features, á=significance level, B=number of permutations, T(per)=counter, F=number of features, PT=permutation probability, P-valuesArray=array of raw P-values

1: Set T(per) to 0
2: df=EstimatedegreeofFreedom()
3: for i   [0, F]
4: 	for j [0, B]
5: 		if B = 0
			T(obs)=EstimatedTestStatistic(V(n,i))
P-value=EstimatedP-value(T(obs)),df)
AdjP-value=AdjustP-value(P-value)
P-valuesArray= P-value;
		else
			V=resample(V(n,i))
			T(obs)'=EstimateTestStatistic(V)
			P-value'=EstimateP-value(T(obs)'),df)
6:		if T(obs) < T(obs)'
			T(per)= + 1
7:	end for
8:	PT=T(per)/B
9: end for
10: AdjustP-valueB&H(P-valuesArray)
11: for i   [0, F]
12: 	if P-value(i) < á
		Output T(obs)(i), P-value(i), AdjP-value(i), PT(i), AdjustP-valueB&H(i)
13: end for

Usage
=====

From windows command console

:\java Btest

Once in the program, the user should provide the following information:

Enter file's name and hit enter: e.g. samples.txt
Enter output file's name and hit enter: e.g. output.txt

What test statistic do you wish to calculate?

 1: Bartlett
 2: t-Test
 3: ANOVA
 4: Chi-square - categorical data e.g. t-Test

How many permutations (R) do you want? e.g. 1000
Significance level (critical P-value e.g. 0.01) ? e.g. 0.05

How statistical tests were calculated
=========================

Bartlett's test


Si2 is the variance of the ith group 
N is the total sample size
Ni is the sample size of the ith group 
k is the number of groups, 
Sp2 is the pooled variance. 

t-Test

Two sample t-test paired data
T=

is the mean difference
Sd is the sample variance
n samples number

Two sample t-test for unpaired data (equal variances)
T=

and  are the samples means 
Sp pool variance

Two sample t-test for unpaired data (unequal variances)

T=

and  are the samples means
S2X S2Y are the samples variances

Chi-square

X2=   
Oi is the observed frequency
Ei expected frequency

ANOVA
Given a Matrix (M), with n samples, p groups, and i,j representing the location of an element in the matrix.
 
			
			
			

SSB = III – I
SSW = II – III
SSTotal = II – I

MSB = SSB/dfB
MSw = SSB/dfw

F = MSB / MSw

Between (B), Within (W)

Testing data sets

Bartlett's test
BartlettTestTable1: 2 classes, 12 samples, 5 and 17 respectively, 8068 features

Chi-square test – categorical  data
ChisquareTestTable1.txt, 3 classes, 33 samples - 10, 12, 11 samples respectively – 334 features 

t-Test – unpaired samples
t-TestTable1.txt: 2 classes, 12 samples - 5 and 17 respectively - 8068 features

t-Test - paired samples
t-TestPaired.txt: 2 classes, 18 samples - 9 and 9 respectively - 1 feature

ANOVA
AnovaTestTable1.txt: 3 classes, 37 samples - 7, 20 and 10 respectively – 14976 features

Output results
==========
The following is an example of the software's output

-----------------------------------Input information-----------------------------

Total number of features: 1
Number of classes: 2
Test statistic: t-Test
Number of permutations: 1000
Significance level: 0.05
------------------------------Permutation results--------------------------------

Feature	test_statistic 	raw_P-value	Adj_P-value	Adj_P-value B&H	Adj_P-value_(PT)
G1	4.12e+00	8.04e-04	8.04e-04	8.04e-04		0.00e+00

Number of features whose P-values were below significance level (0.05): 1
Number of features whose P-value was below significance level according to Bonferroni correction: 1
Number of features whose P-value was below significance level according to Benjamini and Hochberg (B&H): 1
Number of features whose P-value was below significance level according to permutation test: 1

Adj P-value: adjusted P-value, based on Bonferroni multiple testing correction.
Adj P-value B&H: adjusted P-value, based on Benjamini and Hochberg multiple testing correction.
Adj P-value (PT): adjusted P-value, based on permutation test.
-----------------------------------------------------------------------------------
